# Supplementary material for: Optimized PAR-2 RING dimerization mediates cooperative and selective membrane binding for robust cell polarity
Source: EMBO J. 2024 Jun 21;43(15):3214–39. doi: 10.1038/s44318-024-00123-3 (PMC11294563; doi:10.1038/s44318-024-00123-3)
Supplement: Supplementary file 5 — Appendix [file 44318_2024_123_MOESM5_ESM.pdf]

Appendix for

Optimized PAR-2 RING dimerization mediates cooperative and selective membrane binding for robust cell polarity

Authors: Tom Bland, Nisha Hirani, David Briggs, Riccardo Rossetto, KangBo Ng, Neil Q. McDonald, David Zwicker, Nathan W. Goehring

Correspondence: nate.goehring@crick.ac.uk

This PDF file includes:

Appendix Figures S1-S5
Appendix Supplemental Methods - Additional Model Description

Appendix Figures

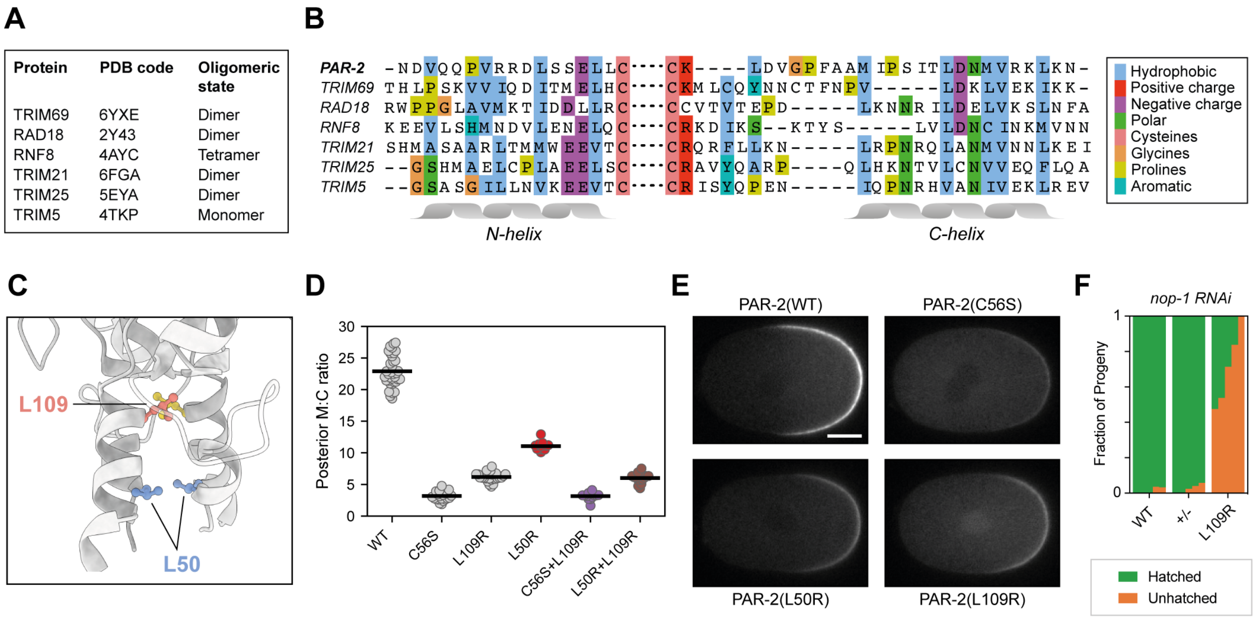

Appendix Figure S1. The PAR-2 RING domain displays the characteristic pattern of hydrophobic residues expected for dimeric RING domains.
(A) RING domains in the Protein Data Bank (PDB) with closest homology to the PAR-2 RING domain (identified using the SWISS-MODEL homology modelling server).
(B) Clustal Omega alignments of the PAR-2 RING domain N and C helices against the RING domains in (A). Note the characteristic pattern of hydrophobic residues (blue).
(C) PAR-2 RING domain dimer structure prediction (AlphaFold) with residues L50 and L109 highlighted.
(D) Quantification of posterior membrane to cytoplasmic ratio for wild type and RING mutant PAR-2. Wild type, C56S and L109R data (grey) repeated from Figures 1 and 2 for comparison.
(E) SAIBR-corrected images of indicated PAR-2 RING variants.
(F) Heterozygous *par-2(ok1723)/+* animals subject to *nop-1(RNAi)* do not exhibit embryonic lethality, indicating that differences in protein dosage cannot account for RING mutant phenotypes. Fraction of unhatched embryos shown for WT (N2, n = 38, 30, 25, 30, 27), *par-2* heterozygotes (+/-, n = 24, 17, 41, 33, 24), and *par-2(L109R)* (L109R, n = 14, 26, 28, 31, 61) animals subject to *nop-1(RNAi)* derived from five independent animals for each condition. Note that heterozygotes express PAR-2 at approximately 60% of WT levels (Rodrigues et al., 2023).

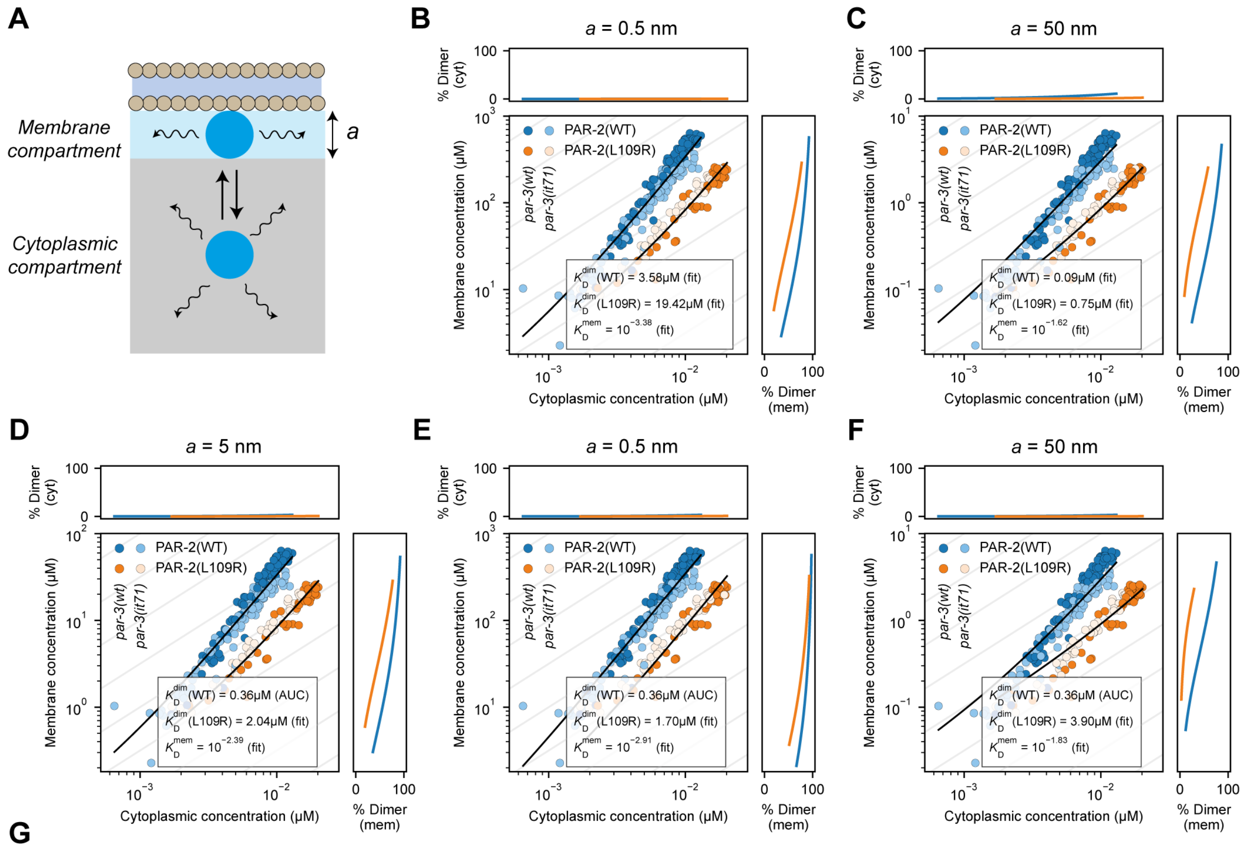

G

a (nm)	K_D^{dim} WT fixed?	K_D^{dim} WT (μM)	K_D^{dim} L109R (μM)	K_D^{dim} fold difference	$\log_{10}(K_D^{mem})$	Figure
5	No	0.425 [0.281, 0.680]	2.460 [1.552, 4.225]	5.79 [5.26, 6.45]	-2.43 [-2.52, -2.35]	3F
0.5	No	3.580 [1.451, 6.567]	19.422 [7.253, 39.020]	5.43 [4.83, 6.16]	-3.38 [-3.50, -3.19]	S6B
50	No	0.095 [0.082, 0.112]	0.750 [0.630, 0.916]	7.90 [7.03, 8.97]	-1.62 [-1.65, -1.60]	S6C
5	Yes	0.358	2.043 [1.870, 2.235]	5.71 [5.22, 6.24]	-2.39 [-2.40, -2.38]	S6D
0.5	Yes	0.358	1.703 [1.565, 1.852]	4.76 [4.37, 5.17]	-2.91 [-2.92, -2.90]	S6E
50	Yes	0.358	3.902 [3.245, 4.871]	10.90 [9.06, 13.60]	-1.83 [-1.85, -1.82]	S6F

Appendix Figure S2. Modelling the plasma membrane as a thin volume compartment.
(A) Schematic of the equilibrium model for membrane association. The system can be described as two volume compartments representing cytoplasmic and membrane-bound states. We assume that diffusion on the membrane compartment is confined to the two-dimensional plane of the plasma membrane, so the membrane compartment can be described as a volume with thickness *a*, where *a* is equal to the diameter of the molecule.
(B) - **(C)** Model fits as in Figure 3F, but using a value for *a* 10x smaller (B) or 10x larger (C).
(D) - **(F)** Model fits for three values of *a* with K_D^{dim} (WT) fixed to the value experimentally determined by AUC.
(G) Table of thermodynamic model parameters. Optimized parameters from six different fits of the thermodynamic model to the in vivo PAR-2(WT) and PAR-2(L109R) rundown data. Shows results with/without fixing the wild type K_D^{dim} to the value experimentally determined by AUC, and with three different values for the protein diameter *a*. 95% confidence intervals determined by bootstrapping.

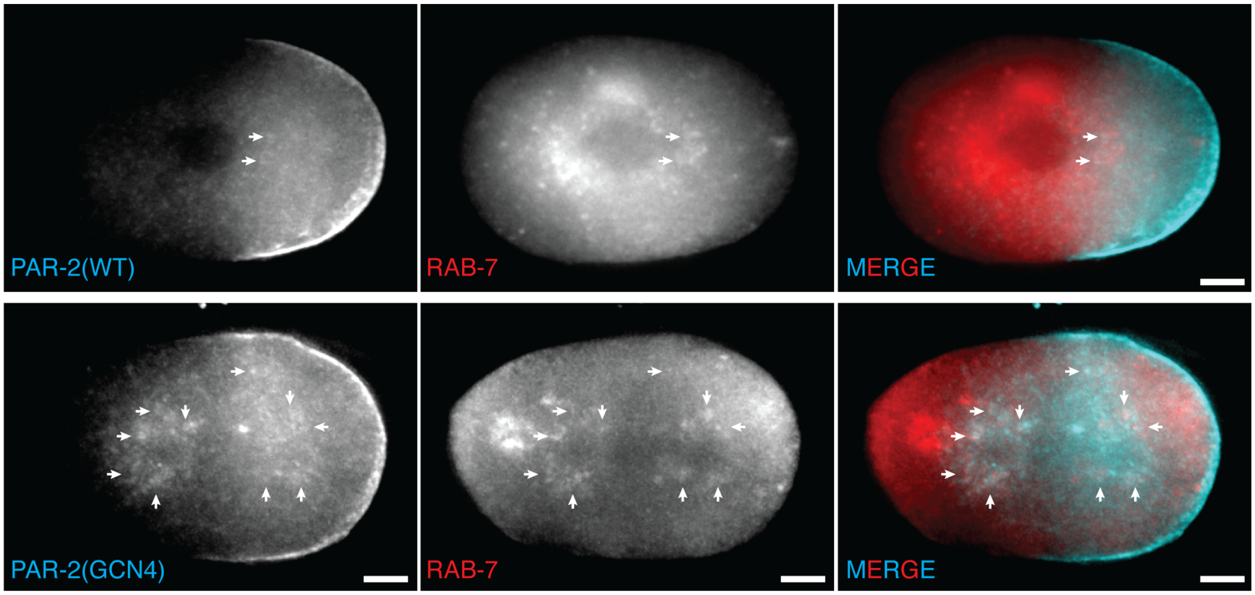

Appendix Figure S3. PAR-2(GCN4) colocalization with RAB-7
Comparison of PAR-2(GCN4) and PAR-2(WT) with RAB-7 in fixed embryos. Single channel and merged images shown. Arrows highlight sample regions with significant overlap. Images are maximum Z-projections of central 10 × 0.25 µm (2.5 µm) sections. PAR-2(GCN4) sample is reproduced at full size from Figure EV3. Note lack of internal PAR-2 positive compartments in wild-type samples. Typical embryos shown.

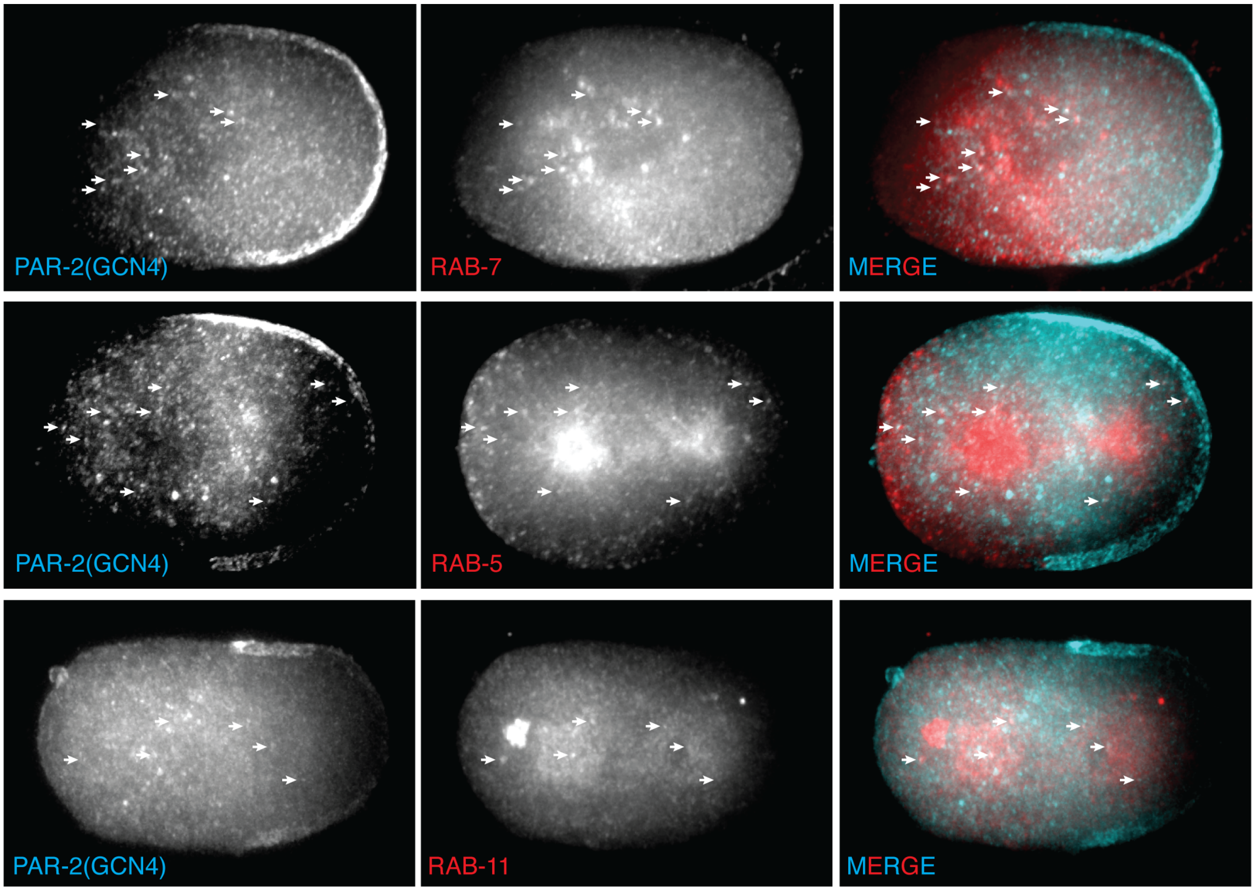

Appendix Figure S4. PAR-2(GCN4) colocalization with various RAB compartments
PAR-2(GCN4) colocalization with RAB-7, RAB-5, RAB-11. Single channel and merged images shown. Arrows highlight sample regions with significant overlap. Images are maximum Z-projections of central 10 x 0.25 μm (2.5 μm) sections. Typical embryos shown.

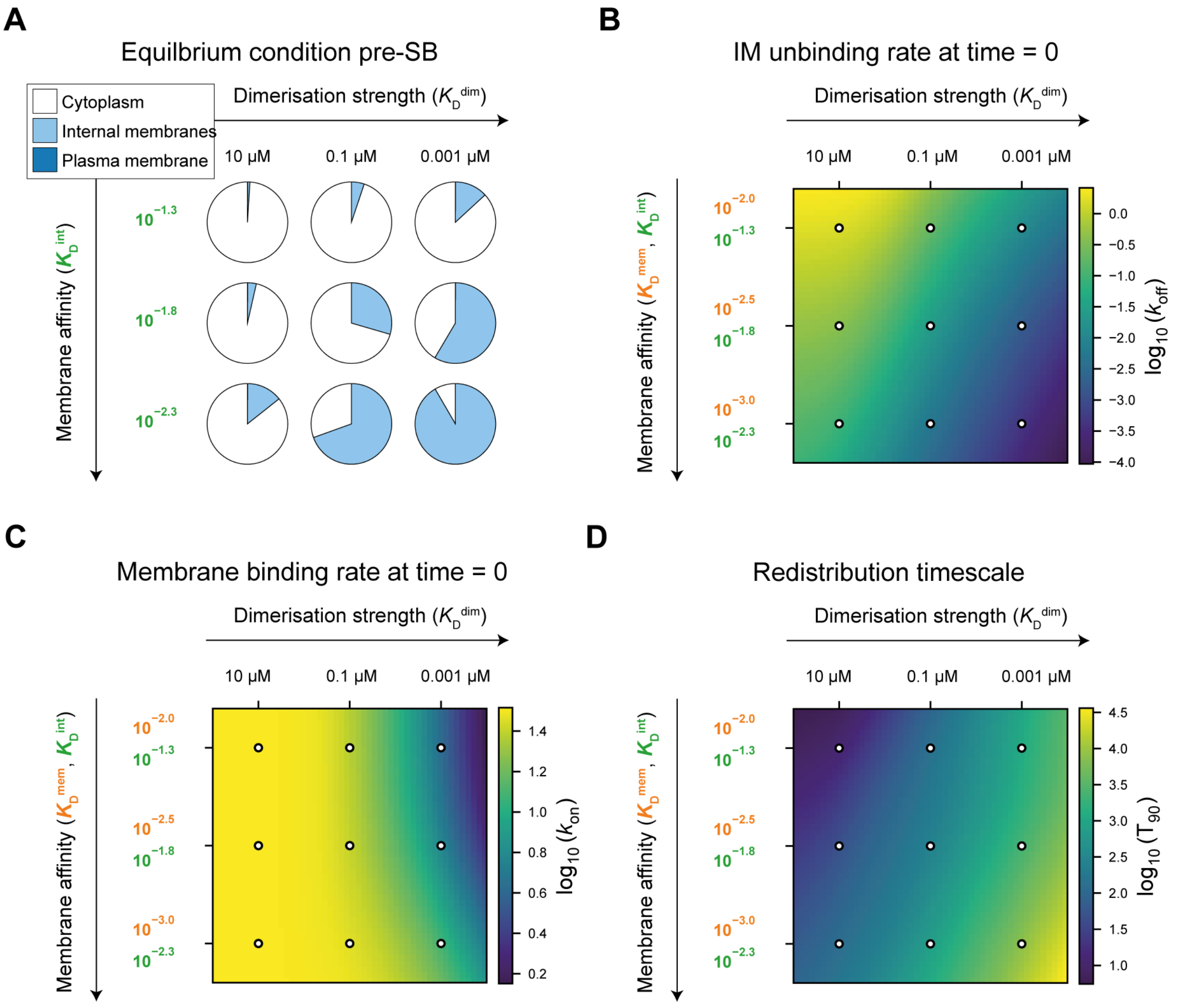

Appendix Figure S5. Entrapment of PAR-2 on internal membranes occurs through dimerization-dependent reduction in membrane exchange rates.
(A) Equilibrium partitioning in the pre-symmetry breaking (SB) equilibrium state, in which PAR-2 is excluded from the plasma membrane.
(B) - **(D)** Starting from a pre-SB equilibrium state, K_D^{mem} is decreased at time=0 to simulate the onset of posterior plasma membrane availability, and systems are followed as they progress towards a new equilibrium state. **(B)** Internal membrane (IM) unbinding rate ($k_{off,n}$) at time $= 0$. White points correspond to the simulations in (A). **(C)** Membrane binding rate (k_{on}) at time $= 0$. **(D)** Redistribution timescale (T90), calculated as the time (in seconds) for plasma membrane concentrations to reach 90% of their post-SB equilibrium level. Note the slowed redistribution kinetics for systems with strong dimerization (low K_D^{dim}) as a result of reduced membrane exchange rates.

Appendix Supplemental Methods

Additional Model Description

1 Introduction

In this document, we provide a detailed description of the thermodynamic approaches used to model PAR-2 membrane association and dimerization. We begin by building the equilibrium model described in Figure 3 of the main text, a four species model with two compartments representing the plasma membrane and cytosol. We then extend this model, firstly to consider the system outside of equilibrium, and secondly to add a third compartment representing internal membranes, both of which form the basis of the analysis in Figure 5 of the main text.

2 Equilibrium Model

We aim to describe the equilibrium behavior of PAR-2, taking into account both dimerization and membrane association. To do so, we begin by building separate thermodynamic descriptions for dimerization and membrane association, before describing a full model which contains both features.

2.1 Thermodynamics of dimerization

First, we consider a non-interacting system containing a mixture of monomeric and dimeric proteins and solvent in a single compartment. In order to study the equilibrium condition, we begin by describing the energetic and entropic contributions, from which we will derive the free energy of the system. From the free energy, we will then derive chemical potentials associated with monomeric and dimeric protein. Then, equating such chemical potentials will provide the dimerization equilibrium condition.

2.1.1 Energy

Consider a system composed of a mixture of monomeric and dimeric proteins and a solvent. Since proteins convert between a monomeric and a dimeric state, the total number concentration of protein is conserved. Thus the following conservation law holds

$$C_1 + C_2 = C_{\mathrm{tot}} \, , \tag{1}$$

where C_1, C_2 are the number concentrations of proteins in the monomer and dimer state, respectively, and C_{tot} is the total number concentration of proteins. (Note that C_2 corresponds to the number of proteins in the dimeric state, not the number of dimers). Since we are considering the system to be non interacting, the energy density of a configuration can be calculated as

$$\frac{H}{V} = RT(c_1\omega_1 + c_2\omega_2) \, , \tag{2}$$

where ω_1, ω_2 are dimensionless internal energies of protein in the monomer and dimer states respectively, V is the total volume of the system (including solvent), $c_i = C_i/N_A$ are the molar concentrations and $R = k_{\mathrm{B}}N_{\mathrm{A}}$ is the gas constant.

2.1.2 Entropy

Starting from the result of Flory (Flory, 1942), we can write the entropy density in terms of number concentrations of protein in monomer and dimer states as follows

$$\Delta s = -k_{\mathrm{B}}\Big(C_1 \ln(C_1\nu) + \frac{C_2}{2}\ln(C_2\nu) + (\frac{1}{\nu} - C_1 - C_2)\ln\left(1 - \nu C_1 - \nu C_2\right)\Big) \, , \tag{3}$$

which depends on the volume of a protein ν. Considering the system to be in the *dilute limit* $\nu(C_1 + C_2) \ll 1$

$$\Delta s = -k_{\mathrm{B}}\Big(C_1 \ln(C_1\nu) + \frac{C_2}{2}\ln(C_2\nu)\Big) \, . \tag{4}$$

Recasting in terms of molar concentrations

$$\Delta s = -R\left(c_1 \ln(c_1 N_A \nu) + \frac{c_2}{2}\ln(c_2 N_A \nu)\right) , \tag{5}$$

which we can rewrite as

$$\Delta s = -R\left(c_1 \ln\left(\frac{c_1}{c_0}\right) + \frac{c_2}{2}\ln\left(\frac{c_2}{c_0}\right) + s_0\left(c_1 + \frac{c_2}{2}\right)\right) , \tag{6}$$

where c_0 is a reference molar concentration introduced to obtain a more familiar form, and $s_0 = \ln(c_0 N_A \nu)$ is a reference entropy introduced by the reference molar concentration.

2.1.3 Free energy and chemical potentials

We obtain the free energy density $f = H/V - T\Delta s$ in terms of molar concentrations of protein in monomer and dimer state, still considering the system to be in the dilute limit

$$\frac{f}{RT} = c_1 \ln\left(\frac{c_1}{c_0}\right) + \frac{c_2}{2}\ln\left(\frac{c_2}{c_0}\right) + c_1(\omega_1 + s_0) + c_2\left(\omega_2 + \frac{s_0}{2}\right) . \tag{7}$$

The associated chemical potentials are then obtained by taking the derivative of the free energy with respect to c_1 and c_2 as follows

$$\mu_1 = \frac{\partial f}{\partial c_1} = RT\left(\ln\left(\frac{c_1}{c_0}\right) + \omega_1 + 1 + s_0\right) \tag{8a}$$

$$\mu_2 = \frac{\partial f}{\partial c_2} = RT\left(\frac{1}{2}\ln\left(\frac{c_2}{c_0}\right) + \omega_2 + \frac{1+s_0}{2}\right) , \tag{8b}$$

where the chemical potentials shown here are chemical potentials associated with one mole of constituents.

2.1.4 Dimerization equilibrium

Considering the dimerization process to be fast, one can assume that dimerization equilibrium is satisfied, i.e.

$$\mu_1 = \mu_2 . \tag{9}$$

Imposing this condition together with the mass conservation condition (1) expressed in terms of molar concentrations

$$c_1 + c_2 = c , \tag{10}$$

we can express the molar concentrations of protein in the monomer and dimer states as functions of the total molar concentration of proteins. We obtain

$$c_1 = \frac{K_D^{\text{dim}}}{4}\left(\sqrt{1 + \frac{8c}{K_D^{\text{dim}}}} - 1\right) \qquad c_2 = \frac{K_D^{\text{dim}}}{4}\left(\frac{4c}{K_D^{\text{dim}}} - \sqrt{1 + \frac{8c}{K_D^{\text{dim}}}} + 1\right) , \qquad \frac{2c_1^2}{c_2} = K_D^{\text{dim}} , \tag{11}$$

upon defining the dimer dissociation constant $K_D^{\text{dim}} = 2c_0 e^{2(\omega_2-\omega_1)-1-s_0}$. Then, inserting these expressions into the free energy expression (7), one obtains an effective free energy depending only on the total molar concentration. Taking the derivative of this free energy with respect to c, the following non dimensionalized effective chemical potential is obtained

$$\frac{\mu}{RT} = \ln\left(\frac{c}{c_0}\right) + (1 + \omega_1) - \frac{1}{2}\ln\left(1 + \frac{4c}{K_D^{\text{dim}}} + \sqrt{1 + \frac{8c}{K_D^{\text{dim}}}}\right) + s_0 . \tag{12}$$

2.2 Thermodynamics of membrane association

We now separately describe the thermodynamics of a protein exchanging between the cytosol and membrane. We describe the system as two compartments: a three-dimensional bulk and a membrane which we describe as three dimensional and of small thickness a, where a is the microscopic length scale of proteins. As protein exchanges between the two compartments, the total concentration is conserved according to the following conservation law

$$c_c + a\psi c_m = c_{\text{tot}} \, , \tag{13}$$

where c_m, c_c are the membrane and cytosol molar concentrations, $\psi = A/V$ is the ratio of membrane surface area over bulk volume, and c_{tot} is the concentration when all the proteins are in the cytosol. To study the equilibrium we proceed as in the previous section and calculate the membrane and bulk chemical potentials. The entropic contribution will now have the same form in the two compartments, so we obtain the following non dimensionalized ideal gas forms

$$\frac{\mu_m}{RT} = \ln\left(\frac{c_m}{c_0}\right) + \omega_m \quad \frac{\mu_c}{RT} = \ln\left(\frac{c_c}{c_0}\right) + \omega_c \, , \tag{14}$$

neglecting the reference entropies since they are equal additive constants, which do not change the behavior, and introducing the non-dimensional internal energies ω_m,ω_c. The equilibrium condition is

$$\mu_m = \mu_c \tag{15}$$

implying a constant ratio of protein in the two compartments at equilibrium according to the difference between the two internal energies

$$\frac{c_m}{c_c} = e^{\omega_c - \omega_m} \tag{16}$$

2.3 Full model

Finally, we describe a full model consisting of both dimerization and membrane exchange. In this model there are four protein states: monomer or dimer, membrane bound or in the bulk. To characterize the four states we *assign the internal energies* as follows

- When a protein is bound to the membrane an energy ω_m is assigned
- When a protein is in dimer state the energy ω_d is assigned

We assume that dimerization and membrane association are independent, i.e. dimerization energy is the same on the membrane and in the cytosol, and the membrane association energy per protein is the same whether the protein is monomeric or dimeric. Writing explicitly the nondimensional internal energies of the four protein species we obtain

$$\omega_{c,1} = 0 \quad \omega_{c,2} = -\omega_d \quad \omega_{m,1} = -\omega_m \quad \omega_{m,2} = -\omega_d - \omega_m \, . \tag{17}$$

Then, imposing dimerization equilibrium in each compartment, we insert the membrane and bulk internal energies into the general effective chemical potential (12), obtaining an *effective two state model* described by the following non dimensionalized membrane and bulk chemical potentials

$$\frac{\mu_c}{RT} = \ln\left(\frac{c_c}{c_0}\right) - \frac{1}{2}\ln\left(1 + \frac{4c_c}{K_{\text{D}}^{\text{dim}}} + \sqrt{1 + \frac{8c_c}{K_{\text{D}}^{\text{dim}}}}\right) + 1 + s_0 \tag{18a}$$

$$\frac{\mu_m}{RT} = \ln\left(\frac{c_m}{c_0}\right) + \ln(K_{\text{D}}^{\text{mem}}) - \frac{1}{2}\ln\left(1 + \frac{4c_m}{K_{\text{D}}^{\text{dim}}} + \sqrt{1 + \frac{8c_m}{K_{\text{D}}^{\text{dim}}}}\right) + 1 + s_0 \, , \tag{18b}$$

where $K_{\text{D}}^{\text{dim}} = 2c_0 e^{-2\omega_d - 1 - s_0}$ and $K_{\text{D}}^{\text{mem}} = e^{-\omega_m}$. For a given total amount of protein c_{tot}, and given values of the dissociation constants $K_{\text{D}}^{\text{dim}}$ and $K_{\text{D}}^{\text{mem}}$, equilibrium membrane and cytosolic concentrations can be calculated according to the equilibrium condition $\mu_m = \mu_c$ and the conservation law (13). (Note that at equilibrium the additive term $1 + s_0$ cancels out so can be neglected). Then, once overall membrane and cytosol concentrations are calculated, concentrations of monomer and dimer within each compartment can be calculated according to the relationship shown previously (11).

3 Kinetic model

To explicitly evaluate membrane exchange rates we study the effective two state model in a non-equilibrium situation. We can imagine bringing this system out of equilibrium, for example placing all the system components in one of the two states, and letting it evolve. As the system relaxes towards equilibrium detailed balance holds (Weber et al., 2019)

$$\frac{s_{\text{on}}}{s_{\text{off}}} = \exp\left(\frac{\mu_c - \mu_m}{RT}\right) , \tag{19}$$

where we define the individual attachment and detachment fluxes s_{on}, s_{off}, that together define the total flux $s = s_{\text{on}} - s_{\text{off}}$. To evaluate the individual fluxes we can use transition state theory (Sneppen and Zocchi, 2005). We can think of a free energy landscape composed of two local minima, corresponding to the membrane and bulk state, separated by an energy barrier. A simple approach is, upon assuming that the metastable states are in a local equilibrium condition and considering the flux small and constant such that it can be considered stationary, to estimate the flux using a Smoluchowski equation. This approach leads to the result that the rate with which components can overcome the energy barrier is proportional to the Bolztmann factor associated with the energy barrier

$$s_{\text{on}} = k \exp\left[-\frac{(\mu_{\text{max}} - \mu_c)}{RT}\right] = \Lambda \exp\left(\frac{\mu_c}{RT}\right) \tag{20a}$$

$$s_{\text{off}} = k \exp\left[-\frac{(\mu_{\text{max}} - \mu_m)}{RT}\right] = \Lambda \exp\left(\frac{\mu_m}{RT}\right) , \tag{20b}$$

where k is a factor related to the kinetic details of the system. We know this to be equal for the two metastable states due to the detailed balance condition (this is not true in general since k depends form the details of the local energy minimum). Together with the Boltzmann factor associated with the local energy maxima, this defines the prefactor Λ. Inserting our expressions for the chemical potentials Eq. (18), we obtain

$$s_{\text{on}} = \frac{\tilde{\Lambda} c_c}{\sqrt{1 + \frac{4c_c}{K_{\text{D}}^{\text{dim}}} + \sqrt{1 + \frac{8c_c}{K_{\text{D}}^{\text{dim}}}}}} \qquad s_{\text{off}} = \frac{\tilde{\Lambda} K_{\text{D}}^{\text{mem}} c_m}{\sqrt{1 + \frac{4c_m}{K_{\text{D}}^{\text{dim}}} + \sqrt{1 + \frac{8c_m}{K_{\text{D}}^{\text{dim}}}}}} \tag{21}$$

where $\tilde{\Lambda} = \Lambda e^{s_0+1}/c_0$. Finally, assuming that these fluxes have a mass action kinetics inspired form $s = k_{\text{on}} c_c - k_{\text{off}} c_m$, we find the concentration dependent rates

$$k_{\text{on}} = \frac{\tilde{\Lambda}}{\sqrt{1 + \frac{4c_c}{K_{\text{D}}^{\text{dim}}} + \sqrt{1 + \frac{8c_c}{K_{\text{D}}^{\text{dim}}}}}} \qquad k_{\text{off}} = \frac{\tilde{\Lambda} K_{\text{D}}^{\text{mem}}}{\sqrt{1 + \frac{4c_m}{K_{\text{D}}^{\text{dim}}} + \sqrt{1 + \frac{8c_m}{K_{\text{D}}^{\text{dim}}}}}} . \tag{22}$$

4 Model incorporating internal membranes

We also explore a model in which proteins have access to a third compartment representing internal membranes. Introducing this compartment leads to the new conservation term

$$c_c + a(\psi c_m + \phi c_n) = c_{\text{tot}} , \tag{23}$$

where c_n is the concentration in the internal membrane compartment and ϕ ratio of internal membrane surface area over bulk volume. As before, we consider the mixture of monomeric and dimeric proteins on internal membranes to be in dimerization equilibrium, obtaining the following chemical potential

$$\frac{\mu_n}{RT} = \ln\left(\frac{c_n}{c_0}\right) + \ln(K_{\text{D}}^{\text{int}}) - \frac{1}{2}\ln\left(1 + \frac{4c_n}{K_{\text{D}}^{\text{dim}}} + \sqrt{1 + \frac{8c_n}{K_{\text{D}}^{\text{dim}}}}\right) + 1 + s_0 , \tag{24}$$

with the internal membrane dissociation constant $K_{\text{D}}^{\text{int}}$. For a given total amount of protein c_{tot}, and given values of the dissociation constants $K_{\text{D}}^{\text{dim}}$, $K_{\text{D}}^{\text{mem}}$ and $K_{\text{D}}^{\text{int}}$, equilibrium concentrations in each of the three compartments are calculated according to the mass conservation term and the new equilibrium condition

$$\mu_c = \mu_m = \mu_n . \tag{25}$$

Evaluating the out of equilibrium exchange rates between the cytosol and internal membranes, we obtain the rate of detachment from the internal membranes as

$$k_{\mathrm{off,n}} = \frac{\tilde{\Lambda} K_{\mathrm{D}}^{\mathrm{int}}}{\sqrt{1 + \frac{4c_n}{K_{\mathrm{D}}^{\mathrm{dim}}} + \sqrt{1 + \frac{8c_n}{K_{\mathrm{D}}^{\mathrm{dim}}}}}\,. \tag{26}$$

References

Flory, Paul J. (1942). "Thermodynamics of High Polymer Solutions". In: *The Journal of Chemical Physics* 10.1, pp. 51–61. DOI: 10.1063/1.1723621. eprint: https://doi.org/10.1063/1.1723621. URL: https://doi.org/10.1063/1.1723621.

Rodrigues, Nelio T.L. et al. (Nov. 2023). *Nonlinear readout of spatial cues underlies robustness of asymmetric cell division*. en. preprint. DOI: 10.1101/2023.11.21.568006. URL: http://biorxiv.org/lookup/doi/10.1101/2023.11.21.568006.

Sneppen, Kim and Giovanni Zocchi (2005). "Physics in Molecular Biology". In: Cambridge University Press. Chap. Kramer's formula Appendix. DOI: 10.1017/CBO9780511755699.

Weber, Christoph A et al. (2019). "Physics of active emulsions". In: *Reports on Progress in Physics* 82.6, p. 064601. DOI: 10.1088/1361-6633/ab052b. URL: https://dx.doi.org/10.1088/1361-6633/ab052b.
