# Supplementary material for: Optimized PAR-2 RING dimerization mediates cooperative and selective membrane binding for robust cell polarity
Source: EMBO J. 2024 Jun 21;43(15):3214–39. doi: 10.1038/s44318-024-00123-3 (PMC11294563; doi:10.1038/s44318-024-00123-3)
Supplement: Supplementary file 6 — Source data Fig. 1 [file 44318_2024_123_MOESM6_ESM.zip › Figure 1/1D/Readme.rtf]

Note raw and AF-corrected (SAIBR) images are provided.
