# Supplementary material for: Optimized PAR-2 RING dimerization mediates cooperative and selective membrane binding for robust cell polarity
Source: EMBO J. 2024 Jun 21;43(15):3214–39. doi: 10.1038/s44318-024-00123-3 (PMC11294563; doi:10.1038/s44318-024-00123-3)
Supplement: Supplementary file 12 — Expanded View Figures [file 44318_2024_123_MOESM12_ESM.pdf]

Expanded View Figures

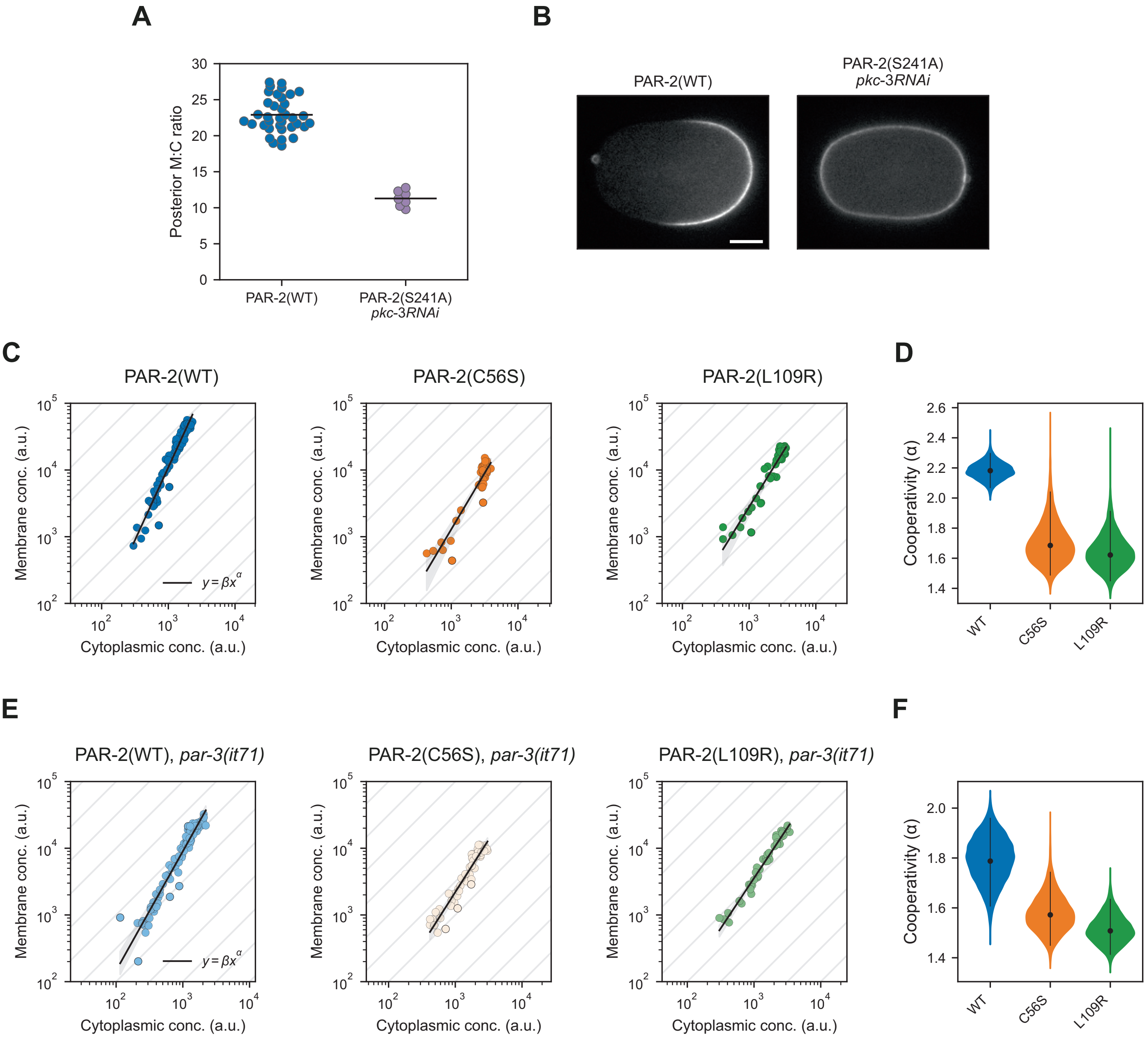

Figure EV1. PAR-2 cooperativity measurements in datasets stratified by polarity state.

(**A**) Quantification of posterior membrane-to-cytoplasmic ratio (M:C) ratio in polarized (WT) and uniform (PAR-2(S241A); *pkc-3(RNAi)*) conditions. Similar to Fig. 1E,I, but PAR-2 is rendered uniform by combining the PAR-2(S241A) mutation that disrupts the key PKC-3 phosphorylation site with *pkc-3(RNAi)*. Combining S241A and *pkc-3(RNAi)* was required to achieve reliably uniform PAR-2 distributions in all embryos. Note PAR-2(WT) data is reproduced from (1E) for comparison. (**B**) SAIBR-corrected images of mNG::PAR-2 in polarized (WT) and uniform (PAR-2(S241A); *pkc-3(RNAi)*) conditions. (**C**) Plots of membrane vs cytoplasmic concentrations of PAR-2 and PAR-2 RING mutants (C56S, L109R) in polarized, *par-3(WT)* cells. (**D**) Probability distribution of cooperativity scores determined from *par-3(WT)* data. (**E**) Plots of membrane vs cytoplasmic concentrations of PAR-2 and PAR-2 RING mutants (C56S, L109R) in unpolarized, *par-3(it71)* cells. (**F**) Probability distribution of cooperativity scores determined from *par-3(it71)* data. Data information: In (**A**), datapoints represent individual embryos. All data shown, mean indicated. (**B**) Scale bar = 10 µm. (**C**, **E**) Black lines show fits to a linear regression model with 95% confidence bands calculated by bootstrapping shown. (**D**, **F**) Best fits to the full dataset (dots) are shown with probability distributions of cooperativity (violin plot) and 95% confidence intervals (lines) calculated by bootstrapping. Additional statistics are available in Table EV2.

A

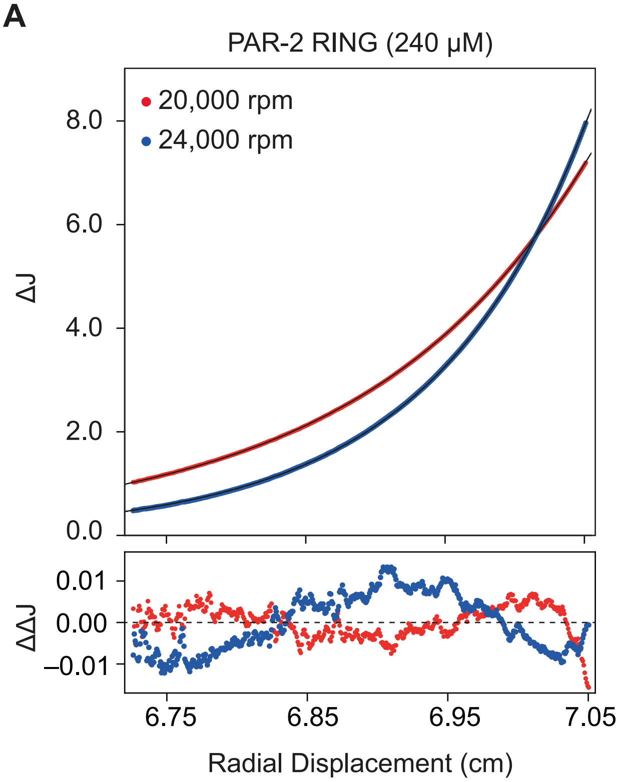

B

Hydrodynamic parameters	
Protein	PAR-2 RING
[a]v (mL.g^{-1})	0.728
[b]ρ (g.mL^{-1})	1.005
[c]η (x10^{2}) (g^{-1} cm^{-1} s^{-1})	1.022
[d]M_r	9,235
[e]ε_{280} (M^{-1} cm^{-1})	1,300
[f]J_{inc} (M^{-1}.cm^{-1})	25,396

[a]Protein partial specific volume; [b]Buffer density; [c]Buffer viscosity [d]Molar mass calculated from the protein sequence; [e]Molar absorbance extinction coefficient; [f]Molar fringe increment.

Sedimentation equilibrium				
PAR-2 RING				
C (µM)	85	125	240	85-240
[a]M_w (kDa)	19.2	18.8	18.0	18-19.2
[b]K_D^{dim} (µM)	0.36	0.37	0.33	0.36
[c]rmsd	0.006	0.006	0.004	0.004 – 0.006
[d]χ^2				1.25

[a]weight averaged molecular weight derived from Global analysis of individual samples using single species model; [b]monomer-dimer equilibrium dissociation constant determined from a global fit using three concentrations and two speeds to a monomer-dimer self-association model; [c]rms deviation observed for each multi-speed sample when fitted individually and globally; [d]global reduced chi-squared from combined fitting of all multispeed data.

Figure EV2. PAR-2 RING self-associates in solution.

(**A**) Multi-speed sedimentation equilibrium profiles determined from interference data collected on PAR-2 RING at 240 µM. Data was recorded at the speeds indicated. The solid black lines represent the global best fit to the data (red, blue points) using a monomer-dimer model (K_D^{dim} = 0.36 µM, reduced χ^2 = 1.25). The lower panel shows the residuals to the fit. (**B**) Full PAR-2 RING sedimentation equilibrium data for AUC performed at multiple concentrations.

 © The Author(s)

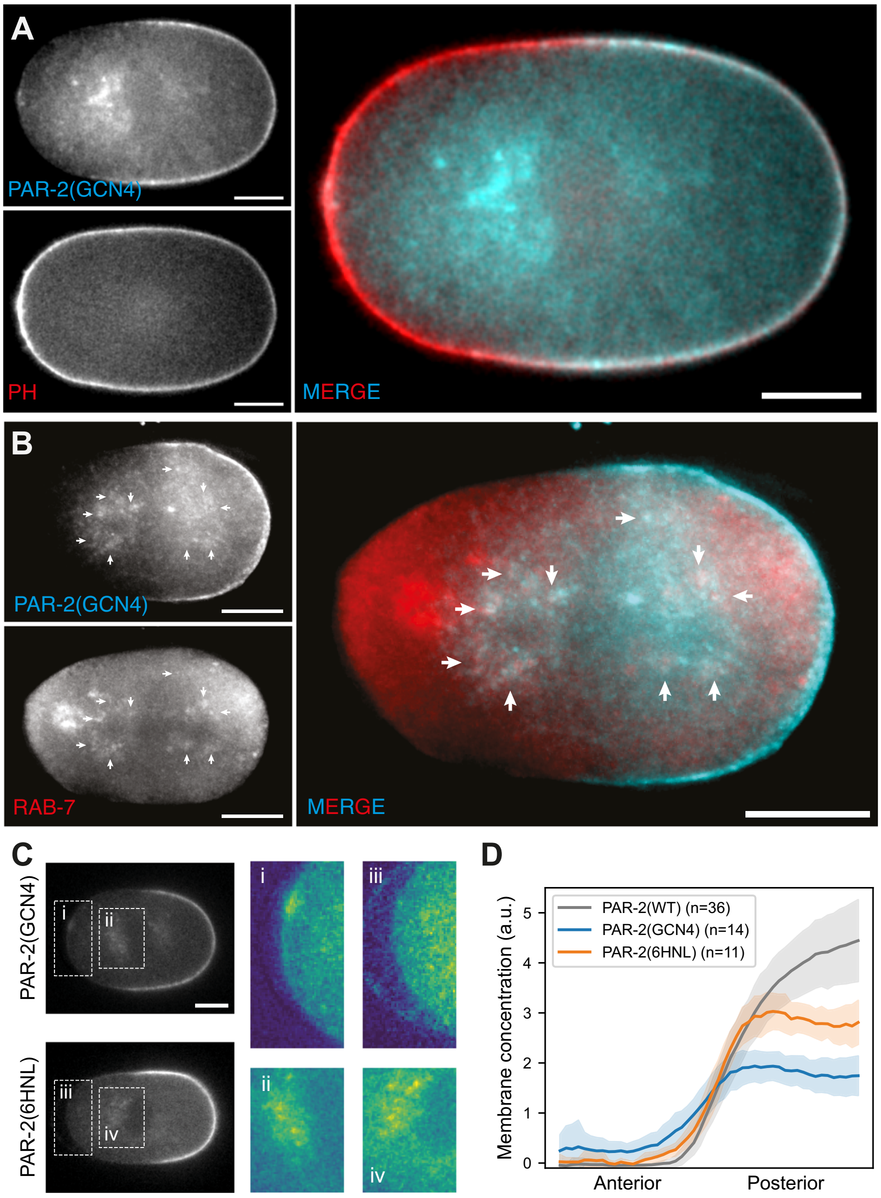

Figure EV3. GCN4-dependent dimerization of PAR-2 leads to recruitment to endosomal membranes, which is recapitulated by an alternative dimerization domain.

(**A**) Midsection confocal images showing no colocalization between mNG::PAR-2(GCN4) and the plasma membrane marker mCherry::PH$_{PLC\delta1}$. Single channel and merged images shown. Typical embryo shown ($n = 4$). (**B**). Colocalization of PAR-2(GCN4) with RAB-7 in fixed embryos. Single channel and merged images shown. Arrows highlight sample regions with significant overlap. Images are maximum Z-projections of central 10 × 0.25 μm sections. Typical embryo shown ($n = 4$). (**C**) SAIBR-corrected images comparing PAR- 2(GCN4) and a version of PAR-2 dimerized via an alternative dimerization domain, 6HNL. Note that PAR-2(6HNL) exhibits similar accumulation on internal membranes and residual signal at anterior membrane. (**D**) Anterior to posterior membrane concentration profiles of PAR-2(GCN4) and PAR-2(6HNL), with PAR-2(WT) shown for reference. Data Information: Scale bars in (**A–C**) = 10 μM. (**D**) Mean ± SD, with number of embryos indicated (*n*).

© The Author(s)

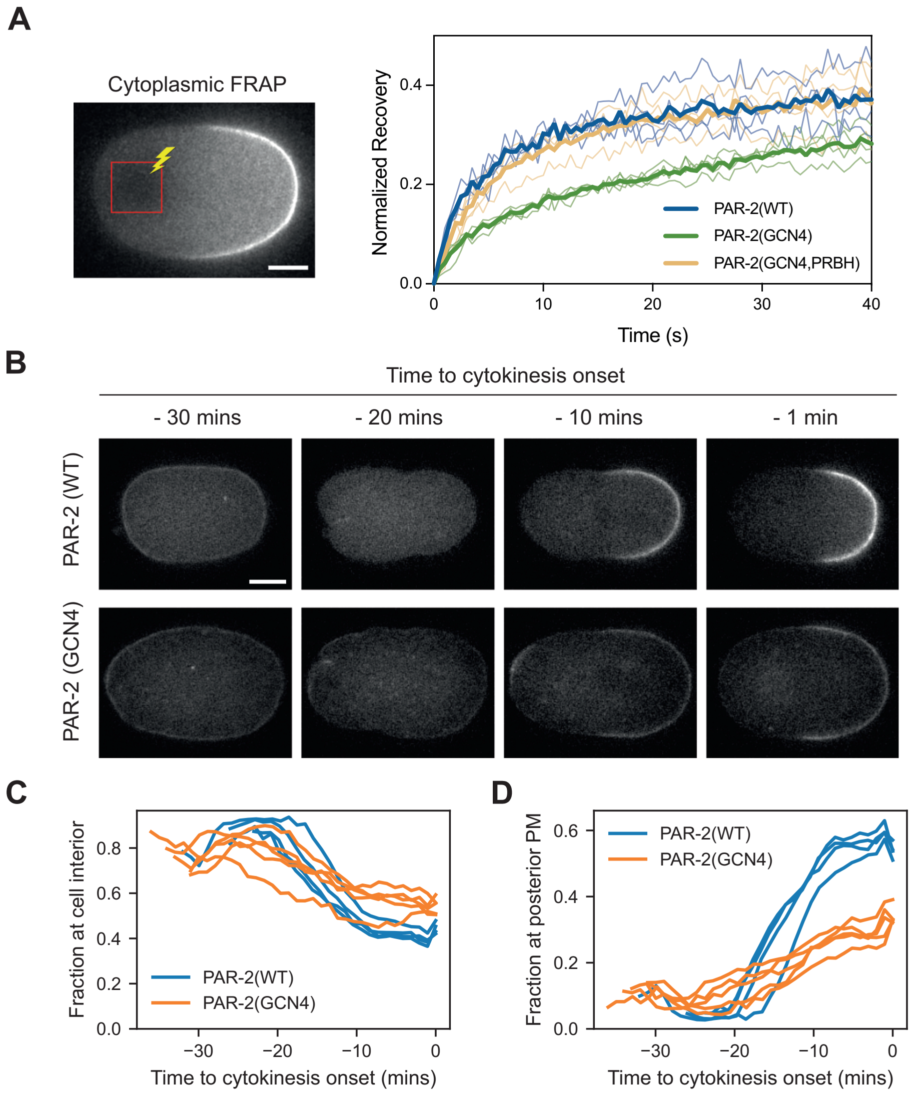

Figure EV4. PAR-2(GCN4) displays reduced dynamics compared to wild type.

(**A**) Normalized recovery curves for cytoplasmic FRAP (see "Methods"). Note that recovery kinetics are reduced for PAR-2(GCN4) compared to wild type, and are restored to near wild-type behavior by mutating residues in the PRBH domain (PRBH). (**B**) Midplane confocal images of PAR-2(WT) and PAR-2(GCN4) localization from meiosis to cytokinesis onset. Together with quantifications in (**C**, **D**), these data show that redistribution from the cell interior to plasma membrane is slowed for PAR-2(GCN4). (**C**) Quantification of total fraction of PAR-2(WT vs GCN4) in the cell interior over time. (**D**) Quantification of total fraction of PAR-2(WT vs GCN4) at the posterior plasma membrane over time. Data information: (**A**) Individual and mean shown. (**C**, **D**) Traces from individual embryos shown. Scale bars in (**A**, **B**) = 10 μm.

 © The Author(s)

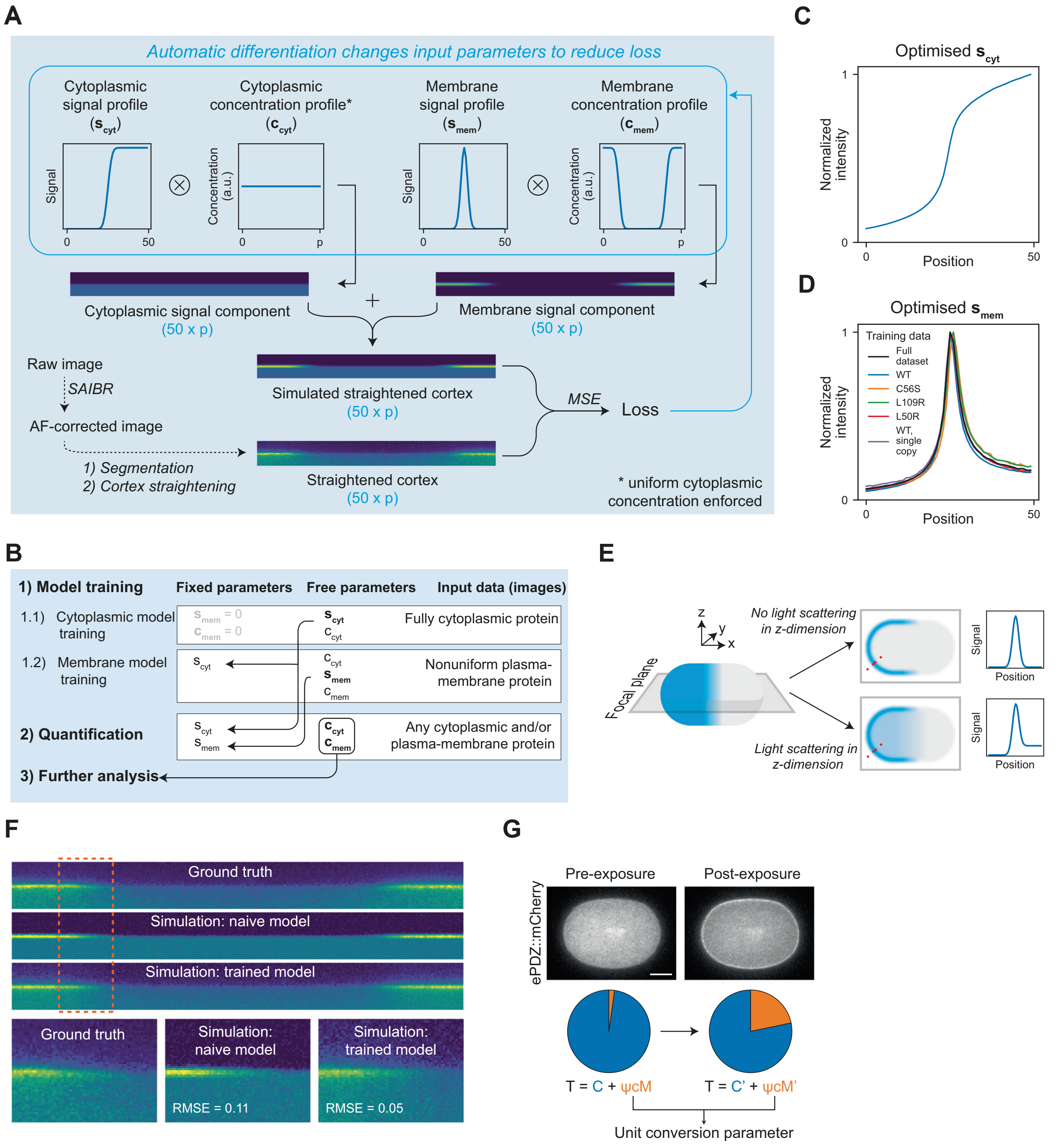
A
Automatic differentiation changes input parameters to reduce loss
Cytoplasmic signal profile (scyt)
Cytoplasmic concentration profile* (ccyt)
Membrane signal profile (smem)
Membrane concentration profile (cmem)
Signal
Concentration (a.u.)
Cytoplasmic signal component (50 x p)
Membrane signal component (50 x p)
Raw image
SAIBR
AF-corrected image
1) Segmentation
2) Cortex straightening
Simulated straightened cortex (50 x p)
Straightened cortex (50 x p)
MSE
Loss
* uniform cytoplasmic concentration enforced
C
Optimised scyt
Normalized intensity
Position
D
Optimised smem
Training data
Full dataset
WT
C56S
L109R
L50R
WT, single copy
B
1) Model training
Fixed parameters
Free parameters
Input data (images)
1.1) Cytoplasmic model training
smem = 0
cmem = 0
scyt
ccyt
Fully cytoplasmic protein
1.2) Membrane model training
scyt
ccyt
smem
cmem
Nonuniform plasma-membrane protein
2) Quantification
scyt
smem
ccyt
cmem
Any cytoplasmic and/or plasma-membrane protein
3) Further analysis
E
Focal plane
No light scattering in z-dimension
Light scattering in z-dimension
Signal
Position
F
Ground truth
Simulation: naive model
Simulation: trained model
RMSE = 0.11
RMSE = 0.05
G
Pre-exposure
Post-exposure
ePDZ::mCherry
T = C + ψcM
T = C' + ψcM'
Unit conversion parameter

Figure EV5. A machine-learning method for extraction of normalized membrane and cytoplasmic protein concentrations from midplane confocal images.

(**A**) Schematic of differentiable model for image quantification. See "Methods" for details. (**B**) Outline of model training and quantification protocol. See "Methods" for details. (**C**) Cytoplasmic signal profile determined by cytoplasmic model training on images of cytoplasmic mNG. (**D**) Membrane signal profiles determined by membrane model training on images of wild-type PAR-2, mutant alleles and single-mNG heterozygotes. Black line shows a model trained on the full dataset. (**E**) Schematic of the expected effects of 3D light scattering on observed midplane signal distributions from membrane protein. (**F**) Example of ground truth (SAIBR-corrected) and simulated images for an mNG::PAR-2(L109R) embryo. Naive model refers to a model in which membrane and cytoplasmic signal profiles are fixed to a Gaussian and error function. Trained model refers to a model in which cytoplasmic and membrane profiles have been trained according to the process outlined in (**B**). Gaussian noise has been added to simulated images to allow for closer visual comparison to the ground truth image. RMSE: root mean square error. (**G**) Optogenetics system used to calibrate cytoplasmic and membrane concentration units. Exposure to blue light promotes an interaction between ePDZ::mCherry and membrane-tethered PH::eGFP::LOV, causing recruitment of ePDZ::mCherry to the membrane. Pie charts show the amount of total ePDZ::mCherry in the cytoplasm, C, and membrane, M, before and after exposure to blue light, which sum to a constant value T. A unit conversion factor (*c*) can be calculated by solving the equations shown, with ψ being the surface:volume ratio. Scale bar = 10 μm.

 © The Author(s)
